# Anti-Mycobacterial Activity of Marine Fungus-Derived 4-Deoxybostrycin and Nigrosporin

**DOI:** 10.3390/molecules18021728

**Published:** 2013-01-29

**Authors:** Cong Wang, Juan Wang, Yuhong Huang, Hong Chen, Yan Li, Lili Zhong, Yi Chen, Shengping Chen, Jun Wang, Juling Kang, Yi Peng, Bin Yang, Yongcheng Lin, Zhigang She, Xiaomin Lai

**Affiliations:** 1 Department of Microbiology, Gangdong Provincial Research Centre for Severe Infectious Disease Prevention and Control Technology, Zhongshan School of Medicine, Sun Yat-sen University, Guangzhou 510080, Guangdong, China; 2 China Ministry of Education Key Laboratory of Tropical Diseases Control, Gangdong Provincial Department of Education Key Laboratory of Functional Molecules from Marine Microorganisms, Zhongshan School of Medicine, Sun Yat-sen University, Guangzhou 510080, Guangdong, China; 3 School of Chemistry and Chemical Engineering, Sun Yat-sen University, Guangzhou 510275, Guangdong, China; 4 School of Pharmaceutical Sciences, Sun Yat-sen University, Guangzhou 510006, Guangdong, China; 5 Department of Pathology, the First Affiliated Hospital of Jinan University (Guangzhou Overseas Chinese Hospital), Guangzhou 510630, Guangdong, China

**Keywords:** anti-mycobacterial activity, 4-deoxybostrycin, differentially expressed genes

## Abstract

4-Deoxybostrycin is a natural anthraquinone compound isolated from the Mangrove endophytic fungus *Nigrospora* sp. collected from the South China Sea. Nigrosporin is the deoxy-derivative of 4-deoxybostrycin. They were tested against mycobacteria, especially *Mycobacterium tuberculosis*. In the Kirby-Bauer disk diffusion susceptibility test, they both had inhibition zone sizes of over 25 mm. The results of the absolute concentration susceptibility test suggested that they had inhibitory effects against mycobacteria. Moreover, 4-deoxybostrycin exhibited good inhibition which was even better than that of first line anti-tuberculosis (TB) drugs against some clinical multidrug-resistant (MDR) *M. tuberculosis* strains. The gene expression profile of *M. tuberculosis* H37Rv after treatment with 4-deoxybostrycin was compared with untreated bacteria. One hundred and nineteen out of 3,875 genes were significantly different in *M. tuberculosis* exposed to 4-deoxybostrycin from control. There were 46 functionally known genes which are involved in metabolism, information storage and processing and cellular processes. The differential expressions of six genes were further confirmed by quantitative real-time polymerase chain reaction (qRT-PCR). The present study provides a useful experiment basis for exploitation of correlative new drugs against TB and for finding out new targets of anti-mycobacterial therapy.

## 1. Introduction

Tuberculosis (TB) remains an international public health burden around the World. Current global estimates indicate that one third of the global population was infected with TB. In 2010 there were still 8.8 million new cases of TB, 1.1 million deaths from TB among HIV-negative people and an additional 0.35 million deaths from HIV-associated TB [1]. China has the largest population account for 20% of the world total. TB remains a big threat to this country. The report of the 4th National Epidemiological Sampling Survey of TB in China stated that the infection rate of TB in the country was 44.5% (about 550 million) [2]. Although currently available drugs kill most isolates of *Mycobacterium tuberculosis*, strains resistant to each of them have emerged, and multiply resistant strains are increasingly widespread, 500,000 of whom are multidrug-resistant (MDR) [3]. The growing problem of drug-resistance combined with the deadly link between TB and HIV infection underscore the urgent need for new anti-TB agents.

**Figure 1:**
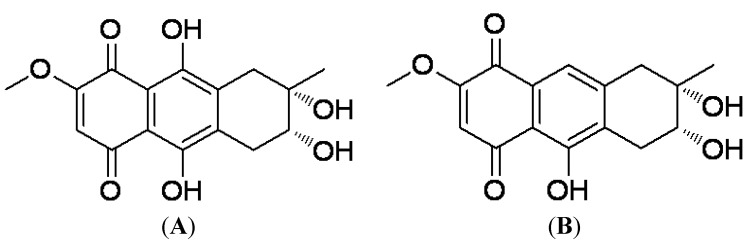
The structure of 4-deoxybostrycin (**A**) and nigrosporin (**B**).

4-Deoxybostrycin (Figure 1A), a natural anthraquinone compound, was first isolated from the fungus *Alternaria eichhorniae* [4]. Its structure was identified by interpretation of spectral data [5,6]. Nigrosporin (Figure 1B) is the deoxy-derivative of 4-deoxybostrycin. It was also a natural product which was first isolated from the fungus *Nigrospora oryzae*. Its structure was identified by interpretation of spectral data [7]. Recently we obtained 4-deoxybostrycin and nigrosporin isolated from the fungus *Nigrospora* sp. [8,9]. It has been reported that 4-deoxybostrycin has various biological properties, including antibacterial [8], phytotoxic [4,10], antimalarial [11], and cytotoxic activities [8,12]. However, its anti-mycobacterial activity is not well investigated.

In this study, we tested it and its derivative’s anti-mycobacterial activity *in vitro*. We first screened and quantitated the anti-mycobacterial activity of the compound and its derivative with the Kirby-Bauer disk diffusion susceptibility test and the absolute concentration susceptibility test respectively. Then we detected the differential expression of *M. tuberculosis* genes after treatment with 4-deoxybostrycin by gene chips and confirmed the results with quantitative real-time polymerase chain reaction (qRT-PCR) experiments. 

## 2. Results and Discussion

### 2.1. Preparation of 4-Deoxybostrycin and Nigrosporin

4-deoxybostrycin and nigrosporin were isolated from the mangrove endophytic fungus *Nigrospora* sp. collected from the South China Sea as previously reported [8,9].

### 2.2. The In Vitro Anti-Mycobacterial Activity of the Compounds

The *in vitro* anti-mycobacterial activity of the compounds was first screened with the Kirby-Bauer disk diffusion susceptibility test. 4-Deoxybostrycin and nigrosporin showed an inhibition zone size of 30 mm and 27 mm, respectively, against *M. bovis* BCG in 4 weeks-cultivated Middlebrook 7H11 agar plates. Their minimum inhibitory concentration (MIC) values against a series of mycobacterial strains were also determined in Middlebrook 7H11 agar slants with the absolute concentration susceptibility test, and these were compared with commercial control drugs including streptomycin (SM), isoniazid (INH), rifampicin (RFP), and ethambutol (EMB). As shown in Table 1, the two compounds both showed inhibitory effects against mycobacteria. Moreover, against two MDR *M. tuberculosis* clinical isolates K2903531 and 0907961, 4-deoxybostrycin exhibited a good inhibition, which even better than that of the first line anti-TB agents. 

2.3. 4-Deoxybostrycin-Induced Alterations in Gene Expression in *M. tuberculosis* H37Rv

According to the study above, 4-deoxybostrycin exhibited a better anti-mycobacterial activity. To find out the differentially expressed genes after treatment with 4-deoxybostrycin and investigate the possible anti-mycobacterial mechanism of the compound at the gene level, we tested the differences of gene expression in *M. tuberculosis* H37Rv just exposed to 4-deoxybostrycin by using the *M. tuberculosis* cDNA microarrays which consists of 3,875 predicted open reading frames of the *M. tuberculosis* H37Rv strain (13). Of all genes tested, 119 genes were significantly different in *M. tuberculosis* H37Rv exposed to 4-deoxybostrycin compared to untreated control. We found that 52 were significantly increased, and 67 were significantly decreased in the 4-deoxybostrycin treatment group. There were 46 functionally known genes, including 24 up-regulated genes and 22 down-regulated genes involved in nucleotide, lipid, energy, coenzyme, carbohydrate metabolism, information storage and processing, and other cellular processes (Table 2).

The tuberculosis cDNA gene chips employed in our study were based on the *M. tuberculosis* H37Rv gene sequence. The functions of these differentially expressed genes included nucleotide, lipid, coenzyme, and carbohydrate metabolism; energy production; information storage and processing; and cellular processes. Therefore, we conclude that 4-deoxybostrycin exerts its anti-TB function through multiple targets. We screened several differentially expressed genes from the compound. 4-deoxybostrycin successfully inhibited drug-resistant *M. tuberculosis* isolates, which allows for new study into the genes affected by the compound to promote the discovery of new anti-*M. tuberculosis* mechanisms and targets. Meanwhile, we can justify and predict the compound’s toxicity and determine if there will be cross-resistance with other anti-TB drugs by examining the affected genes.

### 2.4. 4-Deoxybostrycin-Induced Expression Changes of Some Genes Were Confirmed with qRT-PCR

Total RNA from *M. tuberculosis* H37Rv from either 4-deoxybostrycin-treated or untreated control cultures used for microarray experiments was converted to cDNA, and the expressions of 36 functionally known genes were confirmed with qRT-PCR. Gene expression was normalised with *M. tuberculosis* housekeeping genes. There were only six genes (Rv1518, Rv0282, gnd2, Rv3044 [fecB], Rv3673c, and Rv1372 [pks18]) that were significantly differentially expressed compared to untreated control cultures (Table 3).

We performed qRT-PCR to verify the gene chips. The results showed that their consistency was just 12.3%, indicating that screening through gene chips involves high experimental error that necessitates further confirmation. This array consists of 3,875 predicted open reading frames of the *M. tuberculosis* H37Rv strain [13]. We identified six genes that were significantly differentially expressed compared to untreated control cultures. Gene product of Rv1518 is conserved hypothetical protein, possible glycosyl transferase involved in exopolysaccharide synthesis, was identified in the cell membrane fraction of *M. tuberculosis* H37Rv [14]. Gene product of Rv0282 is identified in the membrane fraction of *M. tuberculosis* H37Rv using 1D-SDS-PAGE and uLC-MS/MS [15], and its function is not clearly understood. Rv3044, probable fecB, FeIII dicitrate-binding periplasmic lipoprotein (see citation below), identified in immunodominant fractions of *M. tuberculosis* H37Rv culture filtrate using 2D-LPE, 2D-PAGE, and LC-MS or LC-MS/MS [13]. Rv3673c, possible membrane protein, thioredoxin-like protein (thiol-disulfide interchange protein) [16]. Rv1372, conserved hypothetical protein, function of which is not clearly understood [17]. The present study provides a useful experiment basis for exploitation of correlative new drugs against TB and for finding out new targets of anti-mycobacterial therapy.

## 3. Experimental

### 3.1. Preparation and Structure of 4-Deoxybostrycin and Nigrosporin

4-deoxybostrycin and nigrosporin were extracted from the fungal fermentation broth by solvent extraction method then purified through chromatography including silica gel column chromatography, ODS column chromatography and HPLC. All reagents and solvents used in experiments were commercially available. Melting points were measured on an X-4 micromelting point apparatus and were uncorrected. IR spectra were measured on a Bruker Vector 22 spectrophotometer (Bruker Corporation, Fremont, CA, USA) using KBr pellets. NMR spectra were determined on a Varian Inova-500 NB spectrometer or Bruker AV-400 NB spectrometer (Bruker Corporation) in CDCl3 or DMSO-*d*6 using TMS as internal standard, and coupling constants (*J*) are in Hz. EI mass spectra were recorded on a DSQ mass spectrometer and ESI mass spectra were obtained on a LCQ DECA XP LC-MS mass spectrometer.

4-deoxybostrycin Red solid (MeOH); mp: 224–225 °C; [α${]}_{D}^{25}$ +90.9 (c 1.1 × 10^−4^, MeOH); ^1^H-NMR (300 MHz, DMSO-*d*_6_): δ 13.18 (s, 1H, 9-OH), 12.62 (s, 1H, 10-OH), 6.42 (s, 1H, 7-H), 4.82 (*d*, *J* = 5.1 Hz, 1H, 2-OH), 4.48 (s, 1H, 3-OH), 3.88 (s, 3H, 6-OMe), 3.61 (dt, *J* = 7.3, 5.1 Hz, 1H, 2-H), 2.82 (dd, *J* = 18.3, 5.1 Hz, 1H, 1-H_b_), 2.77 (d, *J* = 18.1 Hz, 1H, 4-H_b_), 2.64 (dd, *J* = 18.3, 7.3 Hz, 1H, 1-H_a_), 2.56 (d, *J* = 18.1 Hz, 1H, 4-H_a_), 1.18 (s, 3H, 3-CH_3_); ^13^C-NMR (125 MHz, DMSO-*d*_6_): δ 183.39 (C-8), 176.35 (C-5), 161.31 (C-10), 160.38 (C-6), 159.67 (C-9), 138.88 (C-9a), 136.33 (C-4a), 109.47(C-7), 108.97 (C-10a), 106.85 (C-8a), 70.12 (C-2), 68.81 (C-3), 56.95 (6-OMe), 35.72 (C-4), 29.91 (C-1), 25.39 (3-CH_3_); EIMS *m/z* 320 [M]^+^ (100), 302 (41), 287 (30), 259 (61), 247 (96), 234 (20), 219 (45), 205 (10).

Nigrosporin Yellow solid (MeOH); mp: >300 °C; [α${]}_{D}^{25}$ +131.4 (c 1.75 × 10^−4^, MeOH); ^1^H-NMR (400 MHz, DMSO-*d*_6_): δ 12.67 (s, 1H, 9-OH), 7.26 (s, 1H, 10-H), 6.29 (s, 1H, 7-H), 4.76 (d, *J* = 3.6 Hz, 1H, 2-OH), 4.39 (s, 1H, 3-OH), 3.88 (s, 3H, 6-OMe), 3.66 (m, 1H, 2-H), 2.93(d, *J* = 17.5 Hz, 1H, 4-H_b_), 2.85 (dd, *J* = 18.4, 4.9 Hz, 1H, 1-H_b_), 2.77 (d, *J* = 17.5 Hz, 1H, 4-H_a_), 2.69 (dd, *J* = 18.4, 7.1 Hz, 1H, 4-H_a_), 1.17 (s, 3H, 3-CH_3_); ^13^C-NMR (100 MHz, DMSO-*d*_6_): δ 190.76 (C-8), 178.79 (C-5), 161.11 (C-6), 158.31 (C-9), 144.01 (C-4a), 131.70 (C-9a), 127.95 (C-10a), 119.37 (C-10), 110.65 (C-8a), 109.33 (C-7), 70.51 (C-2), 69.41 (C-3), 56.78 (6-OMe), 41.67 (C-4), 29.75 (C-1), 25.07 (3-CH_3_); EIMS *m/z* 304 [M]^+^ (34), 286 (85), 271 (100), 257 (57), 243 (94), 229 (45), 215(54), 201 (23).

### 3.2. Bacterial Strains and Culture Conditions

The following mycobacterial strains were used in this study, *M. bovis* BCG (strain Pasteur, ATCC 35734) and a virulent reference strain of *M. tuberculosis* H37Rv (ATCC 27294) which were obtained from the China Center of Medical Culture Collection; two MDR *M. tuberculosis* clinical isolates (K2903531, resistant to SM, INH, RFP and EMB; 0907961, resistant to SM and EMB), a drug-resistant *M. tuberculosis* clinical isolate (K0903557, resistant to INH), a drug-sensitive *M. tuberculosis* clinical isolate (0907762), and an extensively drug-resistant (XDR) clinical *M. avium-intracellulare* isolate (K0803182, resistant to SM, INH, RFP, LVFX, protionamide and isoniazid aminosalicylate), and *M. avium* reference strain (ATCC 25291) and *M. intracellulare* reference strain (ATCC 13950), which were kindly provided by the Guangzhou Chest Hospital, China.

There were two culture media used in our experiments including Middlebrook 7H9 (Bio-Rad, Richmond, CA, USA) and Middlebrook 7H11 (Bio-Rad). Middlebrook 7H9 broth was used for the cultivation of *M. tuberculosis* H37Rv to test the differential expression of genes *in vitro*. Middlebrook 7H11 was used for the cultivation of all mycobacterial strains in Kirby-Bauer disk diffusion susceptibility test (agar plate) or the absolute concentration susceptibility test (agar slant) to screen or quantify *in vitro* the anti-mycobacterial activity (including MIC) of the compounds.

### 3.3. The Kirby-Bauer Disk Diffusion Susceptibility Test

Bacterial suspension was prepared from 10-days old *M. bovis* BCG or other mycobacterial strain cultures grown on Middlebrook 7H11 agar slant, supplemented with 10% oleic acid, bovine serum albumin (fraction V), dextrose, and catalase (OADC; Remel, Lenexa, KS, USA) and 0.05% Tween 80. The turbidity of the suspension was adjusted to a McFarland no. 3 (9 × 10^8^ CFU/mL) in sterile normal saline. The bacteria was spread on Middlebrook 7H11 agar plates, then discs with 100 μg different kinds of compounds were placed on the plates. All plates were incubated at 37 °C for 4–8 weeks before measuring the diameter of the zone of inhibition of each chemical.

### 3.4. The Absolute Concentration Susceptibility Test

Middlebrook 7H11 agar medium was prepared and sterilized by autoclaving at 121 °C for 15 min. Sterilized medium was supplemented with 10% of sterile OADC at 55–60 °C. The final desired concentrations (100, 60, 50, 40, 30, 20, 15, 10 and 5 μg/mL, respectively, or finer range between 0.1–60 μg/mL) of chemicals were added to respective flasks and poured into 15 mL sterile screwcap plastic test tubes respectively. Upon solidification of medium and preparation of agar slant, the tubes, with or without chemical, were inoculated with 30 μL of 10-fold dilution (3 × 10^7^ CFU/mL) of each mycobacterial strain’s suspension equivalent to a McFarland 1.0 standard (3 × 10^8^ CFU/mL). All the tubes were then incubated at 37 °C for 4–8 weeks. The slants were read when all the cultures had grown on the control slant. If there was no colony grown in the presence of the compound, the lowest concentration of the compound was considered as the MIC.

### 3.5. Determination of the Differential Expressed Genes by Gene Chips and qRT-PCR

#### 3.5.1. RNA Isolation of *M. tuberculosis* H37Rv

Bacterial liquid from the Middlebrook 7H9 medium at log growth period was taken. The experimental group was treated with 4-deoxybostrycin at the final concentration of MIC concentration. The control group was treated with equal volume of solvent which was used to dissolve and dilute the compound. Both groups were incubated for 2 h at 37 °C and then harvested by centrifugation (6000 × g, 5 min). Pellets were resuspend in 95 °C preheated 200 μL MaxBacterial Enhancement Reagent (Invitrogen, Carlsbad, CA, USA) and incubated at 95 °C for 4 min, then transferred to 2 mL screwcap tubes containing 1 ml Trizol reagent (Invitrogen) and 0.5 mL of 0.1-mm-diameter glass beads. Cells were disrupted by strenuous vibration [18]. The RNA was extracted according to the Trizol manufacturer’s instructions.

To remove residual DNA, samples were treated with Turbo DNAse (Ambion, Austin, TX, USA). The integrity of all RNA samples was checked by nondenaturing agarose gel electrophoresis, with RNA concentration quantified by spectrophotometry.

#### 3.5.2. Preparation of Labelled cDNA of Bacteria for Gene Chips and Hybridization

cDNA was synthesized and fluorescently labelled by a direct procedure. The RNA was labelled by Cy5-dUTP in untreated control group and Cy3-dUTP in treated group respectively. After heating at 70 °C for 3 min and chilling on ice, the RNA was reverse transcribed. The labelled cDNA probes were then purified and concentrated using the MiniElute Cleanup kit (Qiagen, Valencia, CA, USA). The total purified cDNA probes were added to the arrays in a hybridization solution containing a final concentration of 5 × SSPE, 2% SDS, 5 × Denhardt’s regent. The arrays were pre-hybridized with denatured salmon sperms DNA at 65 °C for 1 h and then were hybridized overnight at 65 °C, using roller bottles at 6 r.p.m. After removing the hybridized mixture, the chips were washed with preheated Panorama^®^ Microbial Array wash solution (Sigma-Aldrich, St. Louis, MO, USA) according to the manufacturer’s instructions.

#### 3.5.3. Microarray Data Analysis

Microarray slides were scanned using a Typhoon™ FLA 9000 Biomolecular Imager (GE Healthcare, Uppsala, Sweden). Images were processed and the fluorescent intensity of each spot was quantified using the ImageQuant TL v2005 software. Four independent biological replicates were analyzed for each compound group, one swap-dye experiment was included. Median intensity values were corrected by background subtraction. Further analysis was performed using SPSS software (version 16.0 for Windows; SPSS Inc., Chicago, IL, USA). Data was represented by the spot signal as a percentage of the total signal from all spots on the array. Cy5/Cy3 intensity ratios were determined using normalized values. For each gene, the geometric mean was calculated from the intensity ratios of the 2 replicates and the result value was used to determine differences in mRNA abundance between treated group and untreated group. Genes were classified as differentially expressed if the Cy5/Cy3 intensity ratios were out of the range of 0.67–1.5. Statistical significance of the chosen genes was verified by following real-time PCR.

### 3.6. Detection of Differential Expressed Genes by qRT-PCR

Total RNA was extracted from the treated and untreated *M. tuberculosis* H37Rv as mentioned above. cDNA was synthesized from total RNA using random hexamers. The design of primers (Table 4) used for real-time PCR was based on the published genome sequence of *M. tuberculosis* H37Rv [13]. Real-time PCR was performed using SYBR Green (Roche, Mannheim, Germany) in a LightCycler 4800 (Roche, Germany). The end point used in the real-time RT-PCR quantification, Ct, was defined as the PCR cycle number at which each assay target reached the threshold. The data represented the fold change in mRNA expression relative to *M. tuberculosis* incubated with medium alone. To calibrate each amplification result, three *M. tuberculosis* housekeeping genes including 10Sa RNA (http://www.ncbi.nlm.nih.gov/pubmed/1371186), MPT70 (http://www.ncbi.nlm.nih.gov/protein/BAA07184.1) and diaminopimelate decarboxylase (http://www.ncbi.nlm.nih.gov/protein/YP_006514669.1) served as correction factors. The ratio of the same gene expression between the treated and untreated *M. tuberculosis* H37Rv was calculated by the amplified gene copies of 4-deoxybostrycin-treated bacteria divided by the amplified gene copies of untreated bacteria. The criteria used to justify differential expression was the same as above gene chips, *i.e.*, genes were classified as differentially expressed if the ratios were out of the range of 0.67–1.5.

## 4. Conclusions

4-Deoxybostrycin is a natural anthraquinone compound isolated from the mangrove endophytic fungus *Nigrospora sp*. collected from the South China Sea. It showed a good anti-TB activity *in vitro*. Moreover, 4-deoxybostrycin could affect the expression of *M. tuberculosis* H37Rv genes which are involved in nucleotide, lipid, energy, coenzyme, carbohydrate metabolism, information storage and so on. Perhaps 4-deoxybostrycin could be a candidate for the development of new agents to treat TB.

## Figures and Tables

**Table 1:** MIC of 4-deoxybostrycin and nigrosporin to BCG, *M. tuberculosis* and other mycobacterial strains.

Bacterial strains	MIC (μg/mL) ^a^					
	4-deoxybostrycin	Nigrosporin	Control drugs			
			SM	INH	RFP	EMB
*M. bovis* BCG (strain Pasteur, ATCC 35734)	39	15	0.1	0.1	0.05	1.6
*M. tuberculosis* H37Rv reference strain (ATCC 27294)	15	20	0.4	0.05	<0.1	6.4
Clinical MDR *M. tuberculosis* strain (K2903531, resistant to SM, INH, RFP and EMB)	<5	30	>20	>3.2	>20	7.5
Clinical MDR *M. tuberculosis* strain (0907961, resistant to SM and EMB)	10	20	20	0.1	0.1	>6.4
Clinical drug-resistant *M. tuberculosis* strain (K0903557, resistant to INH)	30	30	0.2	2.5	0.25	<1.6
Clinical drug-sensitive *M. tuberculosis* strain (0907762)	10	ND ^b^	<0.1	0.025	10	<1.6
*M. avium* reference strain (ATCC 25291)	>60	>60	20	5	30	3
*M. intracellulare* reference strain (ATCC 13950)	>60	>60	>20	8	>50	2.5
Clinical extensively drug-resistant (XDR) *M. avium-intracellulare* strain (K0803182, resistant to SM, INH, RFP, levofloxacin [LVFX], protionamide and isoniazid aminosalicylate)	>60	>60	0.1	5	6.4	7.5

^a^ MIC (minimum inhibitory concentration) is defined as the lowest concentration inhibiting 100% of the inoculum relative to controls. ^b^ ND: not done.

**Table 2:** Detection of number and functions of differentially expressed *M. tuberculosis* H37Rv genes induced by 4-deoxybostrycin with *M. tuberculosis* cDNA microarray and their functions.

Gene name	Synonym	Mean of ratio	SD	Functional Category
Rv0907	-	0.46	0.03	Cellular processes: Cell envelope biogenesis, outer membrane
Rv1518	-	0.51	0.05	Cellular processes: Cell envelope biogenesis, outer membrane
cmaA1	Rv3392c	1.88	0.08	Cellular processes: Cell envelope biogenesis, outer membrane
Rv1212c	-	1.98	0.21	Cellular processes: Cell envelope biogenesis, outer membrane
fecB	Rv3044	1.66	0.04	Cellular processes: Inorganic ion transport and metabolism
ctpG	Rv1992c	1.60	0.05	Cellular processes: Inorganic ion transport and metabolism
cysA	Rv2397c	1.78	0.08	Cellular processes: Inorganic ion transport and metabolism
Rv0282	-	0.47	0.04	Cellular processes: Posttranslational modification, protein turnover, chaperones
Rv3673c	-	1.78	0.09	Cellular processes: Posttranslational modification, protein turnover, chaperones
Rv2264c	-	1.79	0.02	Cellular processes: Posttranslational modification, protein turnover, chaperones
htpX	Rv0563	1.90	0.15	Cellular processes: Posttranslational modification, protein turnover, chaperones
narL	Rv0844c	0.45	0.10	Cellular processes: Signal transduction mechanisms
Rv3132c	-	1.58	0.04	Cellular processes: Signal transduction mechanisms
Rv1354c	-	2.59	0.25	Cellular processes: Signal transduction mechanisms
ogt	Rv1316c	0.42	0.04	Information storage and processing: DNA replication, recombination and repair
ligB	Rv3062	1.78	0.06	Information storage and processing: DNA replication, recombination and repair
Rv0922	-	1.99	0.21	Information storage and processing: DNA replication, recombination and repair
rpsD	Rv3458c	0.42	0.03	Information storage and processing: Translation, ribosomal structure and biogenesis
rpsS	Rv0705	0.44	0.07	Information storage and processing: Translation, ribosomal structure and biogenesis
prfB	Rv3105c	0.45	0.02	Information storage and processing: Translation, ribosomal structure and biogenesis
rplQ	Rv3456c	0.45	0.03	Information storage and processing: Translation, ribosomal structure and biogenesis
truA	Rv3455c	0.45	0.03	Information storage and processing: Translation, ribosomal structure and biogenesis
infB	Rv2839c	0.46	0.02	Information storage and processing: Translation, ribosomal structure and biogenesis
rplT	Rv1643	0.47	0.04	Information storage and processing: Translation, ribosomal structure and biogenesis
Rv0881	-	1.68	0.07	Information storage and processing: Translation, ribosomal structure and biogenesis
gnd2	Rv1122	0.44	0.03	Metabolism: Carbohydrate transport and metabolism
Rv2039c	-	0.46	0.02	Metabolism: Carbohydrate transport and metabolism
Rv1200	-	1.63	0.04	Metabolism: Carbohydrate transport and metabolism
pgk	Rv1437	1.65	0.07	Metabolism: Carbohydrate transport and metabolism
Rv2471	-	1.83	0.12	Metabolism: Carbohydrate transport and metabolism
Rv2040c	-	2.11	0.19	Metabolism: Carbohydrate transport and metabolism
panB	Rv2225	0.52	0.03	Metabolism: Coenzyme metabolism
Rv1335	-	1.66	0.04	Metabolism: Coenzyme metabolism
nadA	Rv1594	1.73	0.09	Metabolism: Coenzyme metabolism
cobU	Rv0254c	1.78	0.03	Metabolism: Coenzyme metabolism
appC	Rv1623c	0.42	0.05	Metabolism: Energy production and conversion
Rv0247c	-	0.54	0.05	Metabolism: Energy production and conversion
ctaC	Rv2200c	0.37	0.10	Metabolism: Energy production and conversion
Rv1257c	-	1.74	0.04	Metabolism: Energy production and conversion
pks18	Rv1372	0.44	0.04	Metabolism: Lipid metabolism
fadD32	Rv3801c	0.47	0.01	Metabolism: Lipid metabolism
Rv3815c	-	1.77	0.08	Metabolism: Lipid metabolism
deoC	Rv0478	0.53	0.05	Metabolism: Nucleotide transport and metabolism
dgt	Rv2344c	0.59	0.04	Metabolism: Nucleotide transport and metabolism

^a^ Genes were classified as differentially expressed if the Cy5/Cy3 intensity ratios were outside the range of 0.67–1.5.

**Table 3:** qRT-PCR results demonstrating 4-deoxybostrycin-induced differential expression of *M. tuberculosis* H37Rv genes.

Gene names	Rv1518	gnd2	Rv1212c	appC	rpsS	nadA	dgt	prfB	cmaA1	Rv0247c	Rv3815c	Rv0922
Ratio ^a^	0.642	0.539	1.187	0.83	0.793	1.137	1.243	1.01	0.964	0.739	0.933	0.961
	Rv0282	Rv3673c	Rv3132c	cobU	narL	rplT	cysA	rplQ	fadD32	Rv2040c	Rv1354c	Rv1200
	1.989	9.219	0.742	0.919	0.937	0.728	0.805	0.807	1	0.817	0.801	0.924
	Rv3044	Rv1372	Rv1257c	htpX	pgk	ctaC	infB	rpsD	ogt	Rv2264c	Rv0907	Rv1335
	15.46	0.59	1.023	0.846	0.708	1.069	0.787	0.733	0.826	1.275	0.878	1.26

^a^ The ratio was calculated by dividing the amplified gene copies of 4-deoxybostrycin-treated bacteria by the amplified gene copies of untreated bacteria; genes were classified as differentially expressed if the ratios were outside the range of 0.67–1.5.

**Table 4:** The primers of qRT-PCR.

Gene names	PCR product length (bp)	Forward primer (5′→3′)	Reverse primer (5′→3′)
Rv1518	145	gcctcaaccgaaaccacaa	gcgaaagccattccgaca
Rv0282	117	ccaacgcacgcaccactt	cggatgttctcccgcttca
gnd2	111	gccaaaggtggacacgactg	ctgagacaactcacgcaacgag
Rv3044	102	gggtttgacgccgcagtt	ccgacaccacgcaggttatt
Rv3673c	99	cacgatctcgtcggcactg	cgggtcgcaaatgtgatgc
Rv1372	69	ggtggtagtccgcagtagtttc	cgaaataagcgttgagttggtc
Rv0247c	91	ctgttgtagacggaggatgacg	cggagacgaaagctgtggc
cobU	94	cagatgtacctcatcgcagacg	ggtggtgccatcccattctt
htpX	70	gactggcatcctgcgtatcct	gacgtgagacagctcgtggc
rpsS	147	acttcatcggccatacctttgc	gcttgctctttcggtcgtctttt
narL	68	cacgttcaccgagccactca	gcgacgaccacccgttattt
Rv0907	114	acaacgtcgtgacctgggata	cggagcgatgcgagtagag
Rv1200	112	gcttcggcttcgtctacctg	cacagcagtccacccagca
Rv1212c	126	tcgtcggcgtcgtaatgc	gctgggtatcgtaaacctggaa
Rv1335	86	tatccattccgaccatcctgc	gctgatgacggcacccaag
pgk	84	aaggcggacagcattgtgatt	cagcagcgatgtgccaacc
nadA	133	catgttgcaccagcttcgc	ttcgtcggcaccctctacc
Rv2040c	117	caccagcaagatggcgaaca	ggtcccgagacggctacctat
ctaC	137	agccaaacttccaattccactg	caccgtcataccgttcctcatc
Rv2264c	104	gccaccgaagtcgcatacc	agccactgtcactgctccct
dgt	93	tgcgttccaaccgaccct	ccgctgcgttcaataccg
cysA	85	gcgcttacggatcttcaacc	cggattcgtcttccagcacta
infB	132	ccgagtagtagcggatctcca	gcggcattaccgaaaccaa
prfB	119	cgcttgcgttccaacaact	gtgcggctcacccacatt
Rv3132c	94	acaaggctggtcgctgaatg	tcgatggtctggtggaggc
cmaA1	68	tcaggaaacgagcgaaggtg	cgggttgcatccgaaagag
rplQ	92	gaggtcaccgtcttctcccg	ggctacacccgtatcatcaaaa
rpsD	117	acgccgttgacgttgaaatg	actgctgaagatcctcgaaagc
fadD32	107	tcagtccgaagtggcgaaga	gaacctccagcggcaagat
Rv3815c	60	gcctgggatggtccttggt	cgattgtgcccgtcgttgt
ogt	242	cgaaataagcgttgagttggtc	ggcatggctcggtgttga
Rv1354c	441	ctggcggtatgtaggtcttgc	ggcgggtttgttgacttcg
Rv0922	387	ccgcaatgggtttggtcg	ggtggatttggctggaggc
Rv1257c	354	ggtccatgatttcgccgtac	caatacccacccgttgctg
10Sa RNA (control)	85	ttcgctatgcctctgctcg	ggactcctcgggacaacca
MPT70 (control)	135	tgaccagcatcctgacctacc	cggcgttaccgaccttga
diaminopimelate decarboxylase(control)	142	ccttactgctattcgctgtcga	ggcacgggtcacctcactt
